# Genome Sequence of an Environmental Isolate of the Bacterial Pathogen *Legionella pneumophila*

**DOI:** 10.1128/genomeA.00320-13

**Published:** 2013-06-06

**Authors:** Jian Ma, Yongqun He, Bijie Hu, Zhao-Qing Luo

**Affiliations:** Zhongshan Hospital, Fudan University, Shanghai, Chinaa; Unit for Laboratory Animal Medicine, Department of Microbiology and Immunology, University of Michigan Medical School, Ann Arbor, Michigan, USAb; Center for Computational Medicine and Bioinformatics, University of Michigan Medical School, Ann Arbor, Michigan, USAc; Department of Biological Sciences, Purdue University, West Lafayette, Indiana, USAd

## Abstract

We report here the genomic sequence of *Legionella pneumophila* strain LPE509 from the water distribution system of a hospital in Shanghai, China. This is the first complete genome sequence of an environmental *L. pneumophila* isolate. Genomic analyses identified approximately 600 genes unique to LPE509 compared to those of the 7 available *L. pneumophila* genomes.

## GENOME ANNOUNCEMENT

Species of the Gram-negative genus *Legionella* are the causative agent of Legionnaires’ disease. These bacteria are parasites of fresh amoebae in aquatic environments, which are considered the primary source of infection in Legionnaires’ disease outbreaks. Currently, 7 *Legionella pneumophila* genomes are available, all from clinical isolates, i.e., the six serogroup 1 strains 130b (1), Alcoy (2), Corby (3), Lens (4), Paris (4), and Philadelphia 1 (5) and the serogroup 12 strain 570-CO-H (6). These genomic data have provided the information necessary for large-scale comparative genomic analysis, which is instrumental to the study of the evolution of virulence of this important pathogen (7, 8).

To gain insights into the potential differences between clinical *L. pneumophila* isolates and environmental strains, we determined the entire genome sequence of a strain (*L. pneumophila* LPE509) that was isolated from the water distribution system of a hospital in Shanghai, China (9). Genomic DNA was used to prepare 500-bp shotgun libraries to obtain a total of 1,944,445 (94-fold coverage) reads in each library by an Illumina DNA analyzer, which was *de novo* assembled into 36 contigs >500 bp with SOAP*denovo* 1.05. Full assembly was achieved with Geneious by sequencing PCR products that spanned the gaps.

We used the software Glimmer 3 (10) to predict the open reading frames, which were then annotated by BLAST searches against GenBank. tRNA genes and RNA genes were identified with the tRNAscan and RNAmmer programs, respectively, followed by BLAST searches. Results from BLAST searches against known *L. pneumophila* genomes were used to assign gene names. Genome comparison between LPE509 and the other known *L. pneumophila* genomes (strains 130b, 570-CO-H, Alcoy, Corby, Lens, Paris, and Philadelphia 1) was performed with Vaxign (11) and incorporated the ortholog searching program OrthoMCL (12).

The genome of strain LPE509 consists of a single circular chromosome of 3,434,224 bp and a plasmid of 73,490 bp. The average G+C content is 38.32%. It contains 9 rRNA operons, 43 tRNA genes, and 4,285 putative open reading frames, of which 3,246 are predicted genes (75.8%). The genomic organization of LPE509 is highly syntenic with those of the seven already-sequenced *L. pneumophila* strains. Gene clusters encoding all the structural components of the type I secretion system (T1SS), the T2SS, and the Dot/Icm type IVb secretion system (T4BSS) were found in syntenic positions. Components of the type IVb secretion system Lvh were detected in the genome. Interestingly, only five of the proteins LvhA, LvhB11, LvhD4, LvhB4, LvrB, LvrE, LvrC, and LvhB10 are highly similar (*e* value = 1.0E − 10 to 4.0E − 73) to those found in strain Philadelphia 1. LPE509 harbors 274 of the 277 experimentally verified protein substrates in the Dot/Icm transporter (7, 13). The plasmid unique to strain LPE509 carries genes that are putatively involved in resistance to multiple drugs. Compared to the other 7 sequenced strains (130b, 570-CO-H, Alcoy, Corby, Lens, Paris, and Philadelphia 1), LPE509 genes code for 627, 519, 563, 570, 720, 605, and 496 unique proteins, respectively, highlighting the high levels of genomic plasticity among different *L. pneumophila* isolates.

### Nucleotide sequence accession numbers. 

The NCBI accession numbers of strain LPE509 are CP003885 and CP003886.
